# Role of remote ischemic preconditioning against acute mountain sickness during early phase

**DOI:** 10.14814/phy2.12499

**Published:** 2015-08-11

**Authors:** Gaurav Sikri, Anuj Chawla

**Affiliations:** Department of Physiology, Armed Forces Medical CollegePune, India

We read the article titled “Remote ischemic preconditioning delays the onset of acute mountain sickness in normobaric hypoxia” with great interest (Berger et al. 2015). The authors have concluded that remote ischemic preconditioning (RIPC) of 45 min before exposure to normobaric hypoxia transiently reduces symptoms of acute mountain sickness (AMS) and this effect is not associated with reduced plasma levels of reactive oxygen species. The study reports the incidence of AMS as 7% (1 out of 14 subjects) and 21% (3 out of 14 subjects) at 5 and 18 h of hypoxia exposure respectively. AMS was diagnosed based on an AMS-C score ≥0.70 and a Lake Louise Score ≥5. The cut-off values of AMS scoring systems have been debated and studies have supported the use of a combined scoring (Schommer et al. 2012). Since Lake Louise scores of ≥3 (self-report questionnaire) have traditionally been used for labeling an individual as a case of AMS (Roach et al. 1993, 2000), often leading to a doubling of the incidence of AMS when compared to cut-off of score of ≥5 (Dellasanta et al. 2007; Schommer et al. 2012), it would be interesting to know the incidence of AMS at various points of the present study had a LLS of ≥3 been taken as “cut-off” for diagnosing AMS.

It has also been reported that the protective response of ischemic preconditioning is biphasic, with an early phase of protection starting within minutes of the initial ischemic insult and continuing for 2–3 h and the late (or delayed) phase becoming evident after 12–24 h and persisting for 3–4 days (Bolli 2000). In the present study the authors have demonstrated a lack of protection from RIPC against hypoxic damage by assessing the subjects at 18 h. The study from which the historic control group was taken assessed subjects at 8 h post RIPC in addition to the 5th and 18th hour. Had the authors studied their subjects at 8 h post-RIPC as well, and demonstrated a lack of protective effect, the window of no protection between the early and late phase of protection and the temporal profile of protection following RIPC might have been more thoroughly demonstrated.
